# *In situ* fast analysis of cadmium in rice by diluted acid extraction-anodic stripping voltammetry

**DOI:** 10.1039/c9ra03073e

**Published:** 2019-06-26

**Authors:** Jie-qiong Zhang, Ming-hui Zhou, Wei Tian, Yan-xiang Wu, Xi Chen, Song-xue Wang

**Affiliations:** Cereals and Oils Quality and Safety Research Institution, Academy of National Food and Strategic Reserves Administration (Former Name: Academy of State Administration of Grain) No. 23 Yongwang Str, DaXing District Beijing 102600 China wsx@chinagrain.org

## Abstract

In China, the production has not realized intensive cultivation and the problem of cadmium (Cd)-contaminated rice is salient, so it is important to classify rice with different degrees of Cd pollution by rapid detection method *in situ*. This paper established a method with a combination of dilute acid extraction pretreatment and electrochemical devices. Cd was extracted from rice using 3% HCl for 5 min. A standard curve was obtained based on a certified reference material in the rice matrix with different concentrations of Cd, which was fitted with the Cd concentration (μg kg^−1^) against the stripping peak current value (μA), and the linear correlation coefficient was 0.9997. To analyze the applicability of the method, three factors including substrate diluents, particle diameter of the sample, and stability towards the method were evaluated. The limit of detection (LOD) was 2.02 μg kg^−1^, and the repeatability and accuracy were satisfactory. Cd was determined in 142 samples collected from three major grain-producing provinces of China, and the results have good consistence with the microwave digestion-ICP-MS method. The developed method combined dilute acid extraction with a matrix matching standard curve in ASV for the first time, and it was significantly satisfactory for the detection requirements in China.

## Abstract 2

Diluted acid extraction combined with an electrochemical platform achieve analysis on-site in fields or grain depots.

## Graphical abstract

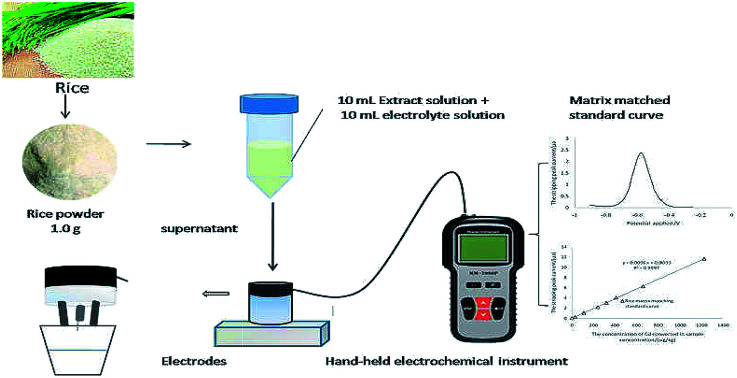

## Introduction

Cadmium (Cd) is a toxic heavy metal that can be accumulated in the body through food, water, and air, and is also associated with the induction of cancer.^1,2^ In recent years, with intensified industrialization and urbanization, agricultural soils polluted with Cd were about 2.786 × 10^9^ m^2^ in China,^3^ and Cd in soil can readily translocate into crops and accumulate in edible grains.^4^ Rice, as the most important staple, supports approximately 65% of China’s population,^5^ so Cd-contaminated rice severely increases the human health risk, and it is extremely urgent to monitor and manage cadmium fluxes of rice in agricultural systems. The most common methods for Cd detection in rice are atomic absorption spectroscopy (AAS), inductively coupled plasma mass spectrometry (ICP-MS), inductively coupled plasma optical emission spectrometry (ICP-OES), and so on.^6–9^ These methods have achieved quick and exact detection, but they are also time-consuming, require sophisticated equipment and are very demanding for the operator, generally in a laboratory setting. Unlike other countries, China’s agricultural production is dominated by scattered smallholders, which also increased a security supervision of difficulty.^10^ The conflicts between small-scale production and large regulation demands are very obvious, so a real-time detection method is necessary when the grain department purchases rice from fields or from grain depots, while the above-mentioned methods are unsuitable for field measurement.

Electroanalytical methods have been extensively employed in recent years, as they are low cost, highly sensitive, easy to operate and portable.^11–13^ In particular, anodic stripping voltammetry (ASV) has been proved to be a high-potential and predominant analytical technique,^14–16^ which is widely applied in the detection of Cd within different complex media, such as tea,^17^ edible oil,^18^ fish tissue,^19^ and soil.^20^ There are numerous reports on Cd detection in rice by ASV, which mainly focused on the choice of metal chelating agent and modification of the electrodes. For example, luminol has been used to chelate Cd and Cu ions in rice samples,^21^ and a crown ether/Nafion-modified screen-printed carbon electrode coated with a bismuth film was prepared to determinate Cd and Pb ions in rice products.^22^ However, the samples which are used for electrochemical detection must be digested completely, so that Cd is released into its ionic form. Conventional digestion methods, such as wet digestion, microwave digestion and dry ashing, need a lot of nitric acid (grade pure), a strong oxidizer, high temperature or pressure, and a long pretreatment time (at least 3 hours).^23^ Therefore, the reported methods were suitable for Cd determination in a lab, but could not be applied to detection in the fields. To resolve the conflicts between the complicated digestion methods and the need for field-portable detection equipment, we established a rapid pretreatment method for Cd in rice using diluted acid without microwave^24^ or ultrasound^25^ assistance, and it could extract Cd from rice at room temperature within 5 min.^26,27^ Sequential extraction has been used to identify the chemical forms of Cd in roots, leaf sheaths, and leaves of both types of rice plants, and 0.6 M of HCl has been used to extract cadmium oxalatefrom frozen shoot and root tissues.^28–30^ However, no studies have been reported on the morphology of Cd in rice grain extracted with diluted acids and its suitability for the detection of electrochemical methods.

**Fig. 1:**
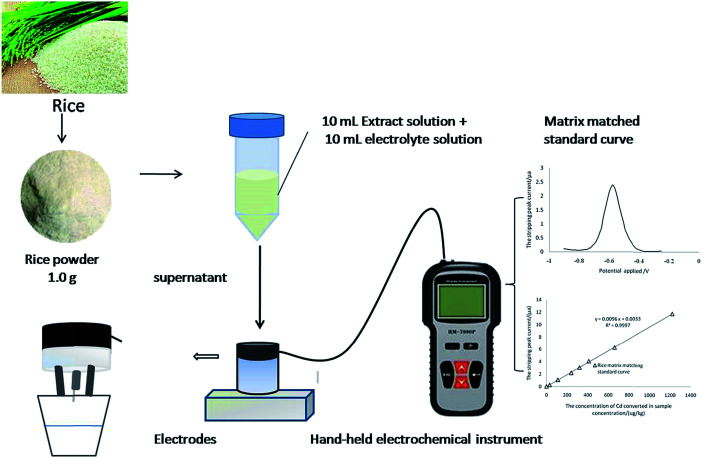
The schematic diagram of the working principle of this method.

The aim of the present work is to develop the *in situ* fast analysis of cadmium in rice. Dilute acid extraction–ASV was developed based on analyzing the form of Cd in the extract of rice and optimizing the conditions of matrix disturbance, dilute acid type, concentration, sample particle size, and detection stability. The schematic diagram of the working principle of this method is shown in Fig. 1. The method was almost “ready-to-use” and can easily be adapted in field testing, and it will provide an alternative way to monitor Cd-contaminated rice in China.

## Experimental

### Testing samples

All certified reference materials (CRM), namely GBW(E)100377, GBW(E)080684a, GBW(E)100350, GBW(E)100348, GBW(E)100349, GBW100361, and GBW(E)100359, were purchased from the National Institute of Metrology, China. IRMM-804 was prepared by the Institute for Reference Materials and Measurements of the European Commission. Reference materials METAL-DJTZK-006 and METAL-DJTZK-007, and other samples containing Cd, were prepared by the Academy of National Food and Strategic Reserves Administration, China.

### Chemicals and apparatus

All reagents were guaranteed grade. Nitric acid, perchloric acid and hydrochloric acid were purchased from Beijing Chemical Reagent Research Institute. Sodium acetate and potassium chloride were supplied by Tianjin Guangfu Science and Technology Development Co., Ltd. All solutions were prepared with double-distilled water. Cd standard solution (1,000 μg mL^−1^, GBW08612) was supplied by the National Institute of Metrology, China. Argon (99.99%) was provided by Beijing North Temperature Gas Factory. A dialysis bag with a molecular weight cutoff of 3500 was purchased from Viskase, USA.

Cd in the extracts was quantified by ICP-MS (7500CX, Agilent, USA) and AAS (ZEEnit 700 P, Analytik Jena, Germany). All voltammetric measurements were conducted by differential pulse anodic stripping voltammetry (HM-5000P, Jiangsu Tianrui, China). The electrode stand consisted of a 3 mm diameter glassy carbon electrode as the working electrode, double-junction Ag/AgCl (3 M KCl, saturated AgCl, and 3 M KCl in the bridge) as the reference electrode, and platinum wire as the auxiliary electrode. A microwave (MARS X, CEM, USA) accelerated reaction system was used for comparison with the sample treatment. A high-speed centrifuge (3-30KS, Sigma Laborzentrifugen GmbH, Germany) was used to separate the extracting solutions.

### Sample preparation

#### Dilute acid extraction

Sample powder (1.00 g) and 10 mL extracting solution (3% HCl, v/v) were transferred into a 50 mL centrifuge tube and then mixed using a vortex mixer for about 5 min. The mixed solution was used for the subsequent voltammetric procedure.

#### Microwave digestion

Samples of grain powder (0.20 g) were digested using concentrated nitric acid (5 mL, 65%, w/w) in a microwave digestion system and then diluted to 25 mL for detection by ICP-MS and AAS. For ASV detection, 0.50 g of grain powder was digested using 7 mL of concentrated nitric acid (65%, w/w). The digestion protocol utilized a 20 min ramp to 180 °C followed by a 20 min hold and 30 min cool down. The digested sample solution for ASV detection was heated at 130 °C until nearly dry, then filtered and diluted to 5 mL for the voltammetric procedure.

### Voltammetric procedure

The same volume of electrolyte solution (59 g of sodium acetate dissolved in 500 mL distilled water) was added into the centrifuge tube containing the mixed solution of dilute acid extraction, and then blended well. To better separate the phases, the mixed solution was centrifuged for 5 min at 10 000 rpm.

The electrode maintenance and analysis parameters were set according to the instructions provided in the electrochemical instrument. The preparation of the glassy carbon surface was carried out as follows: the glassy carbon surface was first polished with polishing paper (Jiangsu Tianrui, China), and the final polishing was carried out using aluminium oxide powder (0.3 μm). The electrode should be rinsed with deionized water between sample measurements.

The instrumental parameters used for ASV were: A constant potential (−1.40 V) was applied for 300 s to form the mercury film. The deposition potential and deposition time were −1.00 V and 110 s respectively. The pre-electrolysis step was followed by stripping from −0.90 V to −0.25 V, which gave a specific stripping current peak in the end.

### Development of the method

#### Development of matrix matching standard curves

Different levels of certified reference materials or reference materials containing Cd were processed as described in the dilute acid extraction and Voltammetry procedure. Matrix matching standard curves were constructed by plotting stripping peak current (μA) against the concentrations of Cd (μg kg^−1^) in the standard samples.

#### Influence of key factors on detection results

Several factors can considerably affect the extraction efficiency and voltammetry procedure. Therefore, the effects of type and concentration of the extracting acid, as well as the effects of sample particle size, settlement method, and number of test times should be investigated to obtain the optimized parameters.

### Evaluation of the detection

#### Detection limit and quantification limit

The LOD and LOQ of the ASV method were calculated as described in ref. 31. The equation is as follows:

1 LOD = 2 × 1.645*S*_b_/*a* = 3.29*S*_b_/*a*

The equation is appropriate for the situation when acquiring voltammetric scans of the analyte-free support electrolyte solution or when using instrumentations which automatically subtract the background from the response of any test sample.

#### Precision and accuracy

The precision of dilute acid extraction–ASV for Cd detection was calculated from 7 consecutive measurements of the sample and expressed as the relative standard deviation (RSD). The accuracy of the method for Cd detection was evaluated using certified reference materials containing Cd at varying concentration levels.

## Results and discussion

### Feasibility of the method

In accordance with the principles of electrochemical measurement, ASV can only be used for testing ions in samples. Whether the speciation of Cd in diluted acid extraction solution could satisfy the requirements of the electrochemical method has become an important basis for the accuracy and stability of the method.

The speciation of heavy metals in the environment (water and soil) and the relationship between bioavailability and toxicity have drawn significant attention in recent years.^32–34^ However, the speciation of heavy metals in food clearly varies from that in the environment, and heavy metals in food exist mainly in the form of macromolecular complexes.^35,36^ Cd in rice is mainly combined with protein to form Cd-binding protein rich charged amino acids or hydrophobic amino acids. The varying chemical forms of Cd in rice grains were also extracted by sequential extraction, and the extraction solution is made up of the following: (i) deionized water, (ii) 5% NaCl, (iii) 70% ethanol, and (iv) 0.2% NaOH.^37,38^ M. H. Zhou *et al.*^26^ previously proposed that an acid reagent at a certain concentration can be used to extract Cd in rice efficiently. They also verified the credibility of Cd extraction using diluted acid by graphite furnace atomic absorption spectrometry (GFAAS).^27^ The dissociation of extracted Cd into the ion form under acidic conditions has yet to be confirmed.

The particle radius of Cd^2+^ is 0.095 nm, however, the molecular mass of the composition formed from Cd with organics is more than 5.5KD.^37–40^ According to this difference, a dialysis bag with an intercept molecular weight of 3500 (the pore size is about 1.5 nm) was used to explore the speciation of Cd in rice. The diluted acid extraction solutions of three certified reference materials pre- and post-dialysis were all digested *via* the microwave digestion protocol then analyzed by ICP-MS. The results are shown in Table 1, and after full dialysis, more than 91% of the Cd could pass through the pores of the dialysis bag. The Cd in rice extracted by diluted HCl mostly was in Cd^2+^ form, which could be detected by electrochemistry.

**Table 1:** Dialysis results of acid extraction solution (*n* = 3)

Sample number	Reference values (μg kg^−1^)	Solid : liquid ratio	Cd concentration (ng mL^−1^)		Ratio of dialysis (%)
			Before dialysis	After dialysis	
GBW(E)100377	261 ± 20	1 : 10	26.3 ± 2.1	2.2 ± 0.1	91.6
GBW(E)100348	240 ± 10		24.4 ± 0.4	0.4 ± 0.1	98.4
IRMM-804	1610 ± 70		164.0 ± 4.0	13.6 ± 0.1	91.7

### Optimization and development of the method

#### Effects of the type and concentration of acid for extraction

The first step of the present study was focused on the optimization of extraction conditions. Initially, the suitable conditions for the stripping peak current signal (μA) were investigated. For this study, we prepared extracted Cd from rice with varying types of acid. Table 2 showed the analytical signal varies with the type of acid. A higher signal was obtained when 1% HCl was employed as the extractant.

**Table 2:** Selection of acid type (*n* = 3)

Acid type	1% HCl	1% HNO_3_	1% HClO_4_
Deposition potential/mv	−592	−574	−578
Stripping peak current/μA	2.37 ± 0.02	1.78 ± 0.04	1.56 ± 0.04

**Fig. 2:**
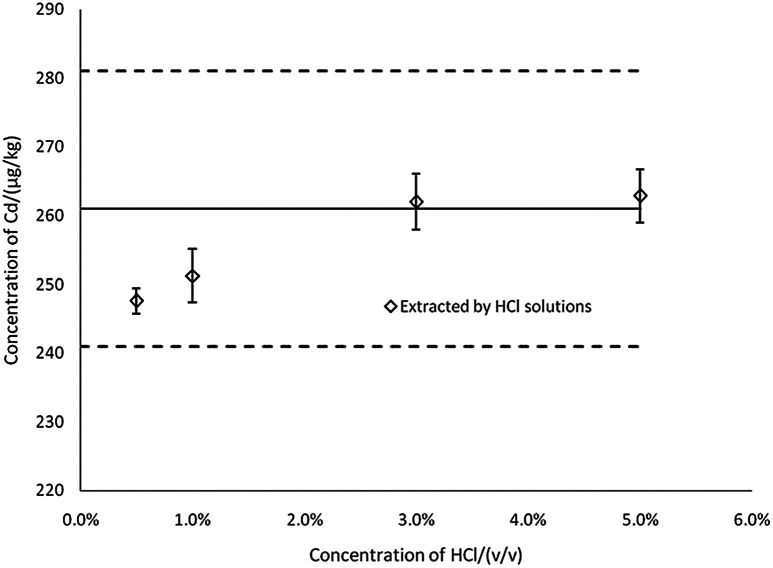
The effects of acid concentration on the determination results (*n* = 3).

**Fig. 3:**
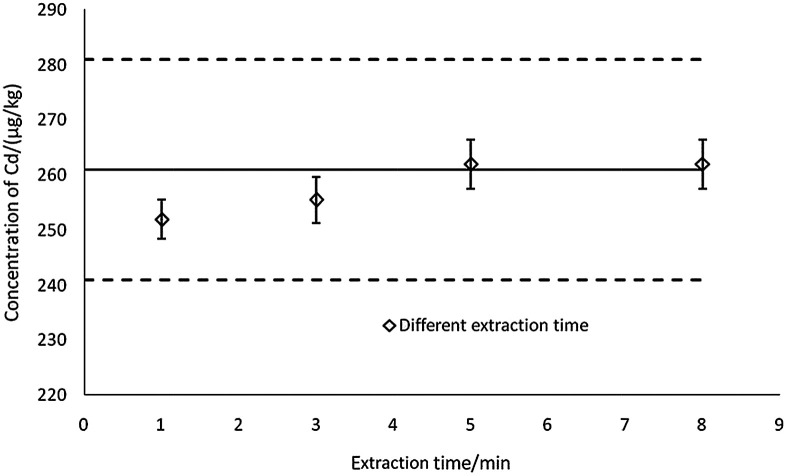
The effects of extraction time on the determination results (*n* = 3).

To evaluate the extraction efficiency at varying concentrations of HCl, the results for Cd in CRM were determined by ICP-MS (Fig. 2). The results showed that the concentrations of Cd in the extract were gradually increased in 0.5–5% HCl (v/v), and when the concentration of HCl was 3% and above, concentrations of Cd in the extract were the most consistent with the certified reference value, *X*_ref_. Thus, 3% HCl was selected as the extraction solution concentration. Note that in Fig. 2 and 3, the thick black lines correspond to *X*_ref_ for Cd of GBW(E)100377, and the dashed lines mark the expanded standard uncertainties of the characterization, *X*_ref_ ± 2*u*_ref_ = 261 ± 20 μg kg^−1^.

#### Effects of extraction time

Other than the optimized conditions, another factor was the extraction time. In Fig. 3, extraction time was varied from 1 min to 8 min, and all of the results were consistent with the certified reference value, *X*_ref_. However, when extraction time was set to 5 min, the test results were closer to the mid-value of the reference value. Thus, 5 min for extracting was more suitable for application.

### Development of the calibration curve

To calculate the content of Cd in samples, the standard curves were constructed with the standard solution of Cd and extracted solutions of reference materials containing different levels of Cd (rice matrix). The abscissa of the curve represented the concentration of Cd (μg kg^−1^), whereas the ordinate represented the stripping peak current (μA). The linear equations of two curves were *y* = 0.0312*x* − 0.4561 and *y* = 0.0096*x* + 0.0033 for matrix matched standard curve and aqueous standard solutions, respectively, and there was a significant difference between the slopes, indicating that the matrix of rice extract exerted a significant effect on signals.

Establishing a correct calibration was essential in any analytical methodology to prevent the occurrence of systematic errors and matrix effects in the procedure. A CRM (GBW10045) was used to investigate the applicability of calibration. Satisfactory results were obtained by using a rice matrix matching the standard, rather than the solution standard (Table 3), which suggested that the matrix matching standard curve could reduce the influence of the rice substrate on the signals.

**Table 3:** Cd contents in GBW10045 as determined by different curves (*n* = 3)

Stripping peak current (μA)	Matrix matching standard curve (μg kg^−1^)	Solution standard curve (μg kg^−1^)	Reference values (μg kg^−1^)
1.84 ± 0.04	194.0 ± 3.50	74.7 ± 1.60	190.0 ± 20.0

**Fig. 4:**
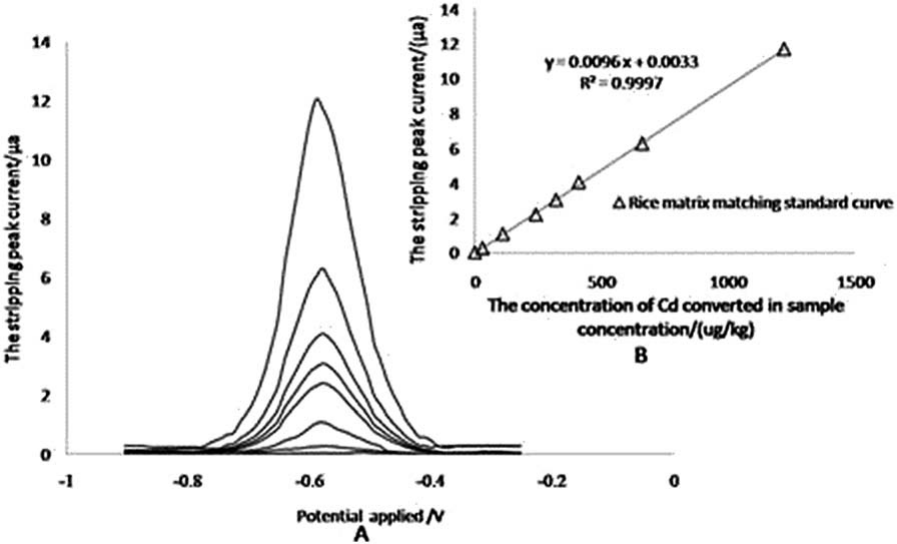
Anodic stripping voltammograms and standard curve. A: anodic stripping voltammograms for extracted solutions of reference materials containing different concentrations of Cd. B: the corresponding fitted linear curve.

The anodic stripping voltammetry for the matrix matching calibration curve was measured by adsorbing Cd^2+^ at specified concentrations for 110 s at −1.0 V, at first, then stripping from −0.90 V to −0.25 V with a linear sweep rate of 0.4 V s^−1^, which gave a special stripping peak at −0.590 V in the end (Fig. 4A). For the matrix matching standard curve, it is difficult to find reference materials with higher concentrations of Cd. The linearity of the calibration plots is verified up to 1220 μg kg^−1^ total concentration for Cd in rice samples (122 μg L^−1^ in extract solution), and the correlation coefficients (*r*^2^) was 0.9997 (Fig. 4B).

## Matrix interferences

**Fig. 5:**
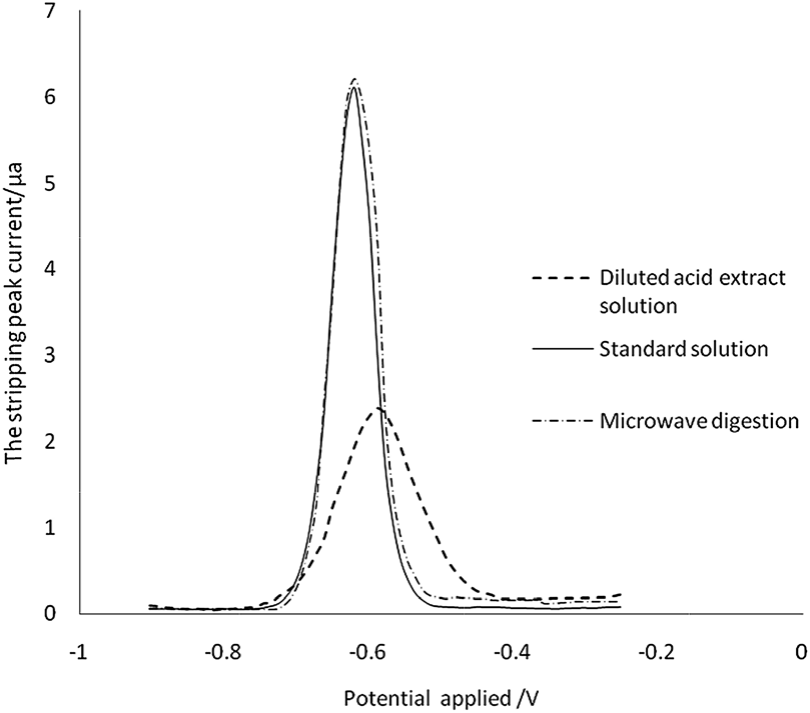
Matrix interferences.

Other metal ions and organic compounds present in the sample solution might co-deposit with the target metal ions, and might result in poor peak resolution. To study interferences, earlier studies have investigated the influence by adding interfering ions, surface-active compounds and EDTA.^41^ However, the compounds from the diluted acid extraction solution cannot be simply simulated by adding the chemicals mentioned above. We therefore directly analyse the ASV results of the sample treated by microwave digestion and diluted acid extraction. As can be seen in Fig. 5, there was no significant difference between the current signals for Cd in the aqueous standard and in the sample digestion solution at the same concentration. The stripping peak of Cd in the sample diluted acid extraction solution was broadened, the stripping current decreased by 65% (from 6.1 μA to 2.1 μA), and the peak potential was shifted from −0.62 V to −0.59 V. These changes indicated that the organic compound in the sample diluted acid extraction solution may be adsorbed on the surface of the working electrode, which reduces the efficiency of the electrode to the target analyte. However, there was no interference to ASV detection when the matrix matched calibration was developed.

### Selection of sample diluents

The matrix interferences were verified to be significant, so the effect of different diluents on the determination of Cd content in rice samples should be evaluated. The diluents were prepared in rice extraction without Cd and aqueous solvent diluents (1 : 1, v/v; extraction solution: electrolyte solution). The influence of two kinds of diluents on the measurement was investigated, and the obtained results are presented in Table 4. The use of aqueous solvent diluents was eliminated as it resulted in noticeably larger values than the reference value, but the blank rice sample was extracted for its satisfactory use for high Cd content samples. This result confirms that the matrix exerts significant effects on this method.

**Table 4:** Determination of IRRM-804 diluted by various sample diluents (*n* = 3)

Dilution ratio	Determination of Cd by ASV (mg kg^−1^)	
	Rice matrix diluent	Aqueous solvent diluent
1 : 2	1.52 ± 0.08	2.08 ± 0.04
1 : 5	1.61 ± 0.05	2.11 ± 0.05
1 : 10	1.59 ± 0.04	2.09 ± 0.06
1 : 20	1.55 ± 0.07	2.14 ± 0.04
Reference value of IRRM-804	1.61 ± 0.07	

#### Effect of sample particle size on detection

**Fig. 6:**
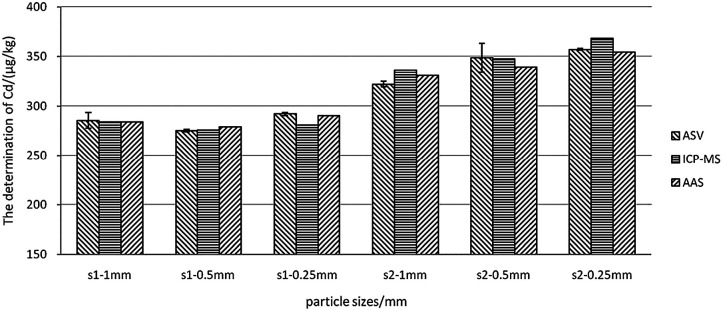
Comparison of the determination results of samples with different particle sizes by ASV, AAS, and ICP-MS. s1 denotes rice sample 1 and s2 denotes rice sample 2.

Sample particle size is another significant factor affecting the extraction efficiency and speed of analysis. It is also an important indicator of the requirements of sample treatment. Thus, the effects of sample particle size on detection have to be evaluated by ASV. Fig. 6 showed that two rice samples with particle sizes ranging from 0.25 mm to 1 mm were analysed by ASV, AAS, and ICP-MS. The results showed no significant difference in varying sample particle size (*P* > 0.05). Therefore, the rice particle size of 1 mm can meet the detection requirements, and this sample processing condition was easily met in the fields.

#### Effect of different sedimentation patterns on the stability of detection by ASV

The state of the mercury membrane is important for detection using ASV.^42^ A complete and uniform mercury membrane directly affects the accuracy and stability of the testing results. However, with the established extraction method, the sample liquid partially contains soluble organic matter and granular material. During voltammetric analysis, the accumulation of stripping and mix scouring processes, and the influence of some organic impurities or particles in the liquid, can result in loss of mercury film, and the mercury films become thinner, rendering them non-detectable.^43–45^ Therefore, the frequency and sedimentation pattern of the extract need to be investigated.

**Fig. 7:**
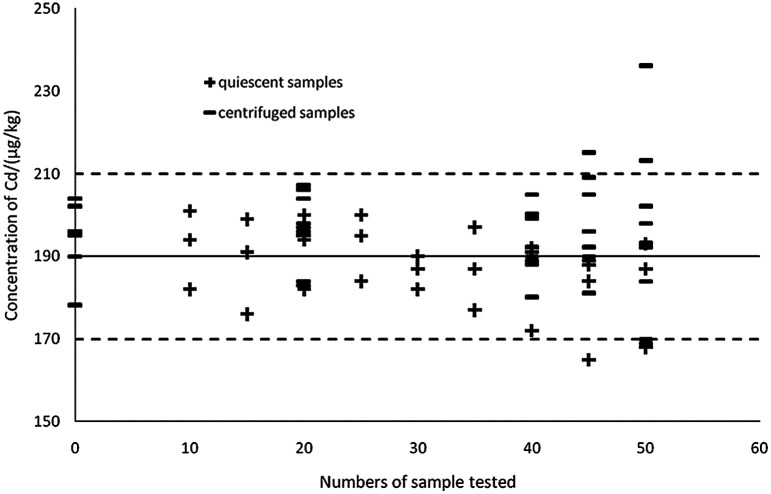
Effects of different sedimentation patterns on detection.

As can be seen in Fig. 7, the thick black lines correspond to *X*_ref_ for Cd in GBW10045, and the dashed lines mark the expanded standard uncertainties of the characterization, *X*_ref_ ± 2*u*_ref_ = 190 ± 20 μg kg^−1^. Sedimentation patterns (centrifugation at 10 000 rpm for 5 min and remaining static for 15 min) showed no difference in detections as the most visible particles in the extractions can sink to the bottom by settling. When using a complete and uniform mercury film, the method can ensure the accuracy of the 40 samples, therefore ASV based on thin-layer mercury electrodes maintains an excellent stability and is highly applied in the present study.^41,46^

### Evaluation of the strategy

#### Limits of detection and quantification

The limits of detection and quantification for the proposed methodology were derived from repetitive analysis of the samples. The detection limit for Cd(ii) was 2.02 μg kg^−1^ (0.20 μg L^−1^ in solution), which was calculated in terms of the 3.29*S*_b_/*a* rule, where *S*_b_ is the standard deviation corresponding to 20 determinations of blank sample detection, and *a* is the slope of the calibration plot. The quantification limit was 6.15 μg kg^−1^ (0.62 μg L^−1^ in solution), according to 10*S*_b_/*a*. The LODs and LOQs of different methods are shown in Table 5. Even though the ASV approach had no advantage in terms of LOD and LOQ, it could fully satisfy the national requirements for the detection of Cd in rice.

**Table 5:** Comparison of LODs and LOQs for different methods

Methods	LOD (μg L^−1^)	LOQ (μg L^−1^)
ASV	0.20	0.62
ICP-MS	0.05	0.17
AAS	0.025	0.085

#### Precision

When the concentration of Cd in rice was 0.235 mg kg^−1^, the precision of dilute acid extraction–ASV (RSD) was 2.16%, obtained by the determination of 7 replicate measurements of the rice sample. This level of precision was satisfactory for this type of procedure.

#### Accuracy

The accuracy of the method was verified by analysis of the CRMs (GBW(E)100378, GBW10045, and IRMM-804). In Table 6, the results are within the assigned range for the CRMs of rice powder and brown rice flour, verifying the accuracy of the proposed method for the determination of Cd in rice.

**Table 6:** Results of ASV detection compared with the values for the reference material (*n* = 3)

CRM no.	Matrix	Results ± SD (mg kg^−1^)	Reference values (mg kg^−1^)
GBW(E)100378	Brown rice	0.164 ± 0.01	0.169 ± 0.015
GBW10045	Rice	0.199 ± 0.01	0.190 ± 0.02
IRMM-804	Rice	1.63 ± 0.04	1.61 ± 0.07

**Fig. 8:**
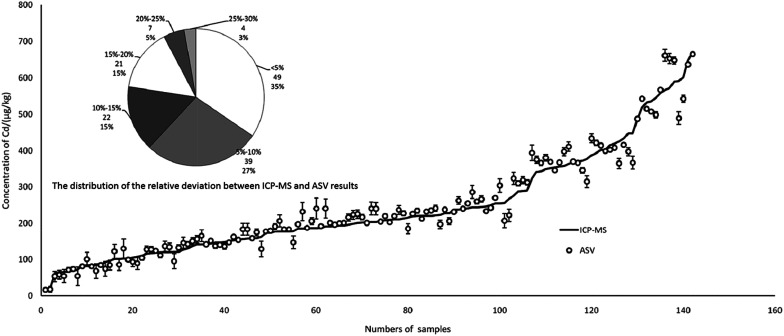
Comparison of Cd results between ICP-MS and ASV.

In total, 142 rice and brown rice samples were collected from three major grain-producing provinces of China, Hunan (*n* = 70), Guizhou (*n* = 38) and Jiangxi (*n* = 34), and the Cd content ranged from 16 to 670 μg kg^−1^ in the samples. As shown in Fig. 8, there was a good relationship between ICP-MS and ASV, which indicated that the results of ASV were credible in real detection. More specifically, the fan diagram showed the distribution of the relative deviation between the ICP-MS and ASV results. The relative deviation below 5% represented the largest portion of total samples (quantity 49, proportion 35%), followed by 5–10% (39, 27%). The relative deviation ranging from 25% to 30% represented the smallest portion of the total samples (4, 3%). In the final analysis, up to 92% of samples could reach a relative deviation below 20% for two different ways.

## Conclusions

We established a rice matrix-matched standard curve coupled with an electrochemical detection method for the determination of Cd in rice and brown rice. Cd was extracted from rice and brown rice using 3% HCl solution. The pretreatment of samples could be completed within 5 min, and then the extracted solution was determined by ASV. The whole procedure was easily accomplished with 15 min. The detection limit of the method was 2.02 μg kg^−1^. Precision and accuracy tests indicated that the proposed diluted acid extraction–ASV method could be used in the analysis of Cd in rice samples. Moreover, compared to the previous methods for the detection of Cd in rice and brown rice by ASV,^21,22^ the pretreatment method needs less time, less corrosive acid of high concentration, and no instruments for high temperature and pressure. Meanwhile, the device for detection using ASV is portable, and easy and simple to operate, so that it could meet the urgent needs for the rapid determination of Cd, particularly in the field of grain purchase or supervision in countries with serious Cd contamination.

## Compliance with ethical standards

### Funding

This study was funded by the research project of public welfare grain industry (Grant No. 201513006) and the research project of National Special Project for Key Science and Technology of Food Safety (Grant No. 2017YFC1601300).

### Ethical approval

This article does not contain any studies with human participants or animals performed by any of the authors.

### Informed consent

Not applicable.

## Conflicts of interest

There are no conflicts to declare.

## Supplementary Material
